# Likelihood Ratio Approach and Clinical Interpretation of Laboratory Tests

**DOI:** 10.3389/fimmu.2021.655262

**Published:** 2021-04-16

**Authors:** Walter Fierz, Xavier Bossuyt

**Affiliations:** ^1^ Schweizerischer Verband der Diagnostikindustrie (SVDI), Bern, Switzerland; ^2^ Clinical and Diagnostic Immunology, Department of Microbiology, Immunology and Transplantation, KU, Leuven, Belgium; ^3^ Immunology Service, Department of Laboratory Medicine, University Hospitals, Leuven, Belgium

**Keywords:** clinical interpretation, laboratory tests, likelihood ratio, harmonization, quality control

## Introduction

Laboratory tests are an important component in the diagnostic process. From an analytical point of view, most tests have reached high technical standards resulting in quantitative results with very high precision and accuracy. The challenge for the clinician then is how to interpret those results. It is particularly difficult when different test systems use different scales and arbitrary units for a given biomarker, as is often the case in immunologic testing. For the clinician it is demanding to estimate the predictive value of a diagnostic test result. A solution to this problem that is advocated here is to provide likelihood ratios as a measure of the predictive value of test results. This approach is not only useful to harmonize interpretation between assays and assay platforms but can be employed as well in external quality control programs. However, the concept of likelihood ratios in clinical diagnostics, although not new, is not yet generally accepted and needs further promotion by demonstrating its usefulness.

Some 55 years ago, a “*technic for the estimation of the predictive value of diagnostic test results in the subject tested when the sensitivity and specificity of the test and the prevalence of the disease in the population are known*” was described (1). At that time, the technic was limited to dichotomous, qualitative test results. Later, the approach has been extended to intervals of test results and their likelihood ratio (LR) (2–6). The LR of a diagnostic test result is defined by its likelihood in diseased subjects (sensitivity) versus non-diseased subjects (1-specificity). In the field of autoimmunity, test result interval-specific LRs have been applied for the diagnosis of rheumatoid arthritis (7, 8), vasculitis (9, 10), systemic rheumatic diseases (11–16), inflammatory bowel disease and celiac disease (17–22).

It has been realized that expressing results in the form of LRs provides a convenient way to harmonize test results which otherwise would be expressed in various units and provider-defined scales, making it difficult to compare results. This has led to a proposal for harmonization of anti-neutrophil cytoplasmic antibody (ANCA) testing (23, 24), antinuclear antibody testing (25, 26) and autoimmunity tests in general by reporting test result-specific LRs (27, 28). The calculation of LRs of test result intervals has been further extended to arbitrary quantitative test results (29, 30) and applied, for example, for the diagnosis of Alzheimer’s disease (31), ANCA testing (24), antinuclear antibody testing (26) and celiac disease (22).

For the clinician, LRs could be a valuable diagnostic measure (32–35). Nevertheless, a wide application of LRs in diagnostic laboratory testing is not observed today. This might have different reasons, such as:

- a LR is related to a specific diagnosis and, habitually, the clinician does not inform the testing laboratory on the precise diagnostic question.
- a test might be used for screening purposes resulting in a differential diagnosis.
- there is a dearth of data on LRs (and consequently laboratories do not report LRs).

With regard to the differential diagnosis, it should be noted that LRs for each differential diagnosis are very valuable to estimate the relative weight of possible diagnoses (36, 37). Establishing LRs needs clinical studies to be performed, either by the *in vitro* diagnostics industry, the laboratories, or a collaboration of both. As this has a cost, reimbursement of laboratory tests should consider the additional clinical value of the diagnostic information given by the LR (38), which is not the case today.

## What Needs to Be Done?

**Figure 1:**
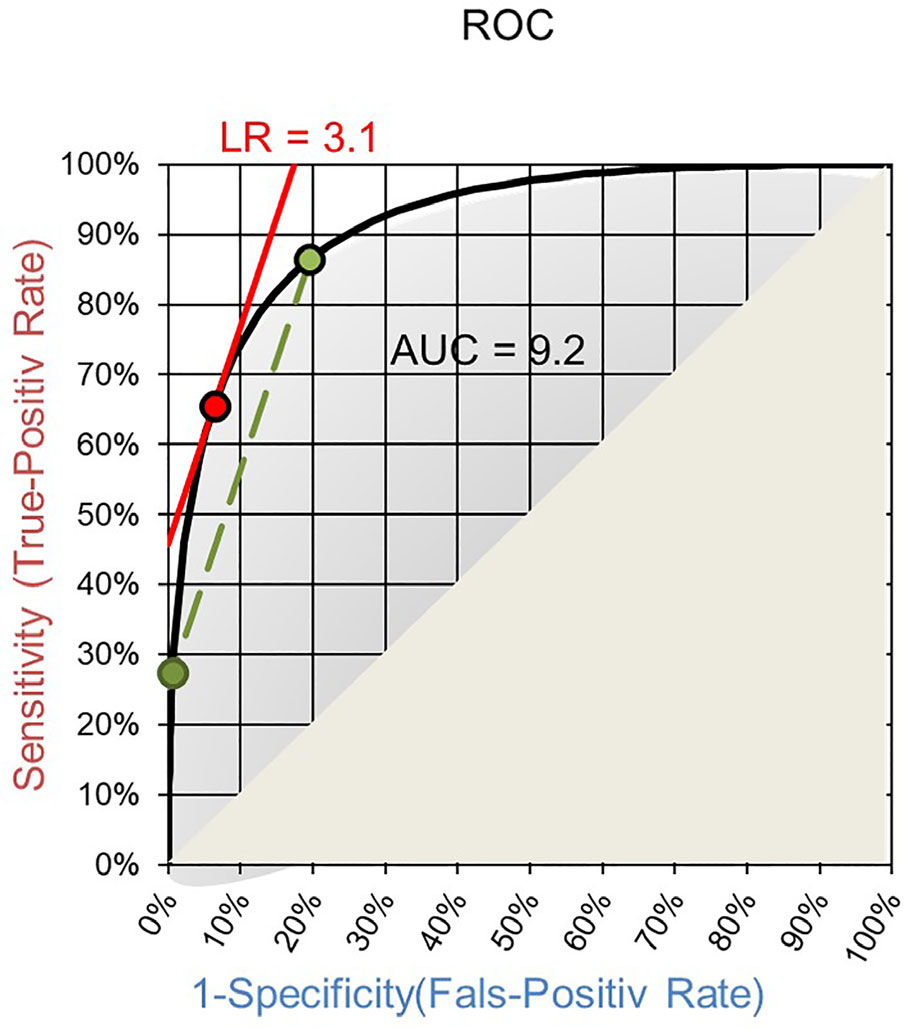
ROC curve with AUC. The slope of the secant (green) gives the LR of an interval of test results and the slope of the tangent (red) for a specific quantitative test result.

The field will benefit from applying LRs as quantifiable diagnostic values of laboratory tests and as means for harmonizing otherwise incompatible quantities of test results. The Receiver Operating Characteristics (ROC) curve of a test is a good basis for establishing LRs. Such ROC curves are routinely established to choose a cut-off for qualitative readouts and for calculating the area under the curve (AUC). On ROC curves the LR of a test result interval is given by the slope of the corresponding secant to the curve between the two endpoints of the interval (**Figure 1**) (39). Making the interval smaller and smaller the LR of a single test result is reached as the slope of the tangent to the ROC curve at the point corresponding to the test result (**Figure 1**) (39).

Since the AUC expresses the discriminant power of a test, the test producer has a high interest to publish such ROC curves. Usually only the graphical display of the curve or even only the AUC and the cut-off are published, but not the test result values corresponding to the individual points of the curve. Some publications shared the complete ROC curve dataset, which allowed to calculate the LRs using the Bézier curves method (31). Based on published ROC curves on fasting capillary glycemia testing (40), D-dimer testing (41), PSA testing (42), HbA1c testing for gestational diabetes mellitus (43), and an Alzheimer’s test (44), we determined test-result specific LRs. These data are given in **Supplemental Data Figure 1**.

Having access to the raw data of clinical studies and the LRs, the next step will be to guide the clinicians to understand the use of LRs. One way certainly is to apply LRs in differential diagnosis. As an example, when performing antinuclear antibody tests (ANA) for screening for connective tissue disease one would get different LRs for different diseases. This would allow the clinician to weigh the suspicions derived from other clinical data. Based on published data on antinuclear antibody testing (45), we deduced the titer-specific LR for the various systemic rheumatic diseases. The results are shown in **Supplemental Data Figure 2**.

Another advantage of using LRs is the harmonization of different techniques, scales, units etc. (24). It certainly would make it easier for the clinician to interpret one single scale, namely LR, than having to get acquainted with different titers, units/ml, ug/ml, mmol/l etc. Even tests using the same scale are not always comparable between different test producers but could be harmonized with LRs. Clinical guidelines giving clinical decision limits for certain test results could improve on such harmonized LRs, not only for dichotomous readouts (46, 47), but also for quantitative results.

LRs have a direct function in estimating the probability of a diagnosis. According to Bayes’ theorem the pretest odds multiplied by the LR of the test result give the posttest odds. Now, the clinician in daily practice may not be used to thinking in such numbers of probability but would rather develop an intuition for them. Nevertheless, when it comes to explain, defend, and document a diagnostic decision, LRs would be very helpful. Estimating the pretest odds might be the more difficult part. Starting from the prevalence of the disease in the population to which the patient belongs, the clinician usually adds the anamnestic and clinical findings leading to the use of a laboratory test in order to include or exclude the suspicion. A low suspicion would need a much higher LR for inclusion than a high suspicion and, conversely, a high suspicion would need a much lower LR for exclusion than a low suspicion. For example, when testing healthy pregnant women for HIV-infection the pretest odds would be around 1:100’000. Receiving now a positive screening test from the laboratory a confirmation would of course be necessary, which usually needs a second blood sample. But what should the doctor tell the patient in the meantime? Above what level of screening test results is the LR starting to get higher than 1? HIV-Screening tests have a very low cut-off to reach a maximal sensitivity, but this leads to the fact that low screening results have an LR smaller than 1. The same holds for anti-nuclear antibody screening by indirect immunofluorescence. A low titer positivity (e.g.) 1:40 or 1:80 has a low LR (<1) for systemic rheumatic disease (14).

In daily practice, the clinician probably is not thinking in terms of pretest probabilities or even pretest-odds. However, the clinical experience provides a level of premonition for a diagnosis that should be confirmed or refuted by the laboratory test. To what extend such change of suspicion is valid depends of course on the quantitative level of the test result. For standardized and frequently used tests, the clinician would intuitively have a feeling for how much the quantitative test result assures the diagnosis. But often, especially in non-harmonized test systems and when the result is at a level near the cut-off point between positivity and negativity, the information content of the result will be overestimated and therefore misleading. As an example, we recently defined for 8 different ANCA test systems assay-specific test results that corresponded to a LR of 0.1, 1, 10 and 30 (24). For the different assays, the test result that corresponded to a LR of 10 was 35 Units, 48.5 CU, 8.6 IU/mL, 2.8 AI, 10 IU/mL, 13.8 U/mL, 48 U/mL and 10.7 IU/mL (24). All these values have the same clinical meaning, namely that the chance to find such value is 10 times higher in patients with ANCA-associated vasculitis than in individuals without an ANCA-associated vasculitis. The provision of LR values would give the individual results a meaning without knowing the scales and cut-offs. When LR values will be reported by the laboratories, together with the quantitative results, the intuitive diagnostic estimation of the clinician will get with time a new dimension that is generally applicable, independent on the specific test. The diagnostic information provided by a LR of 3, 10, 30 or 100 will get a semantic content on how much secure the clinician can be in the daily routine, without calculating probabilities.

Another example that we recently worked out is on antinuclear antibodies (ANA). Lately, platforms that measure fluorescence intensities have been introduced into clinical laboratories. We defined the light intensity units that corresponded to a LR of 0.1, 0.33, 1, 3 and 10 for the NovaView, an automated ANA system from Inova Diagnostics. By doing so we found that the light intensity unit that corresponded to a LR of 0.1 was very close to the cutoff for positivity proposed by the company (26). This means that values that correspond to the cutoff are 10 times more likely to be found in individuals without an ANA-associated rheumatic disease than in patients with an ANA-associated rheumatic disease (which was in agreement with the many false positives reported by the clinicians). We report the LRs for ANA-associated rheumatic disease associated with the ANA fluorescence intensities, which helps the clinician with interpreting test results. One could even go a step further and define pattern-specific LR. Indeed, we demonstrated that the positive predictive value of ANA depends on the pattern, with the highest positive predictive values for the centromere pattern (48).

Finally, we also associated LRs to tissue transglutaminase antibody levels and this revealed that cutoffs are not aligned between manufacturers (22). Here again, test result specific LRs could help to align results between manufacturers.

A further aspect in using LRs by the laboratory is that it can be applied in external quality control. It is nowadays standard for clinical laboratories to take part in external quality controls. When starting to provide LRs of test results to the clinicians it would be important to also compare LRs with other laboratories. Upcoming differences would probably rather have their origin in the different specifications of clinical studies used to establish the ROC curves than in the technical procedures in the laboratory. This would be important to find out to improve harmonization of tests. It might lead to harmonize clinical diagnosis.

## Conclusion

We here presented the concept of LR and illustrated its application in autoimmune serology. There are several advantages in applying LR to communicate the diagnostic value of a test. It allows to report test result- (or test result interval)-specific information and to harmonize interpretation between assays and assay platforms. It can not only be applied for specific diseases, but also in differential diagnosis. The concept can also be employed in external quality control programs. The advantages of using LRs in autoimmune serology is being recognized by experts and *in vitro* diagnostic companies and using LR has been proposed by international organizations (EASI, EFLM, …) as a convenient way to harmonize ANCA test results. Major efforts still need to be done in order to get the concept more generally accepted and applied.

## Author Contributions

The authors contributed equally to the manuscript. All authors contributed to the article and approved the submitted version.

## Conflict of Interest

The authors declare that the research was conducted in the absence of any commercial or financial relationships that could be construed as a potential conflict of interest.

## Supplementary Material

Supplementary Figure 1 ROC curves with test result values (o) corresponding to the individual points of the curve (left) and LR as a function of test results (right) as calculated by the Bézier curves method (29). Test results with LR=1 are indicated in red. **(A)** Fasting capillary blood glucose as a screening test for diabetes (40). **(B)** D-dimer testing for suspected pulmonary embolism in outpatients (41). **(C)** PSA testing Gleason grade ≥7 vs Gleason grade <7 or no cancer (42). **(D)** HbA1c Test as a Tool in the Diagnosis of Gestational Diabetes Mellitus (43).

Supplementary Figure 2 Based on published immunofluorescence test results (45) LRs are calculated for positive results (> 1:160) LR^+^, borderline results (1:40- 1:160) and negative results (<1:40) LR^-^ in systemic lupus erythematosus (SLE), Sjogren syndrome (SS), systemic sclerosis (SSc), dermatomyositis and polymyositis (DM/PM), mixed CTD (MCTD) and rheumatoid arthritis (RA).
