# Supplementary figures and images for: Likelihood Ratio Approach and Clinical Interpretation of Laboratory Tests
Source: Front Immunol. 2021 Apr 16;12:655262. doi: 10.3389/fimmu.2021.655262 (PMC8086426; doi:10.3389/fimmu.2021.655262)

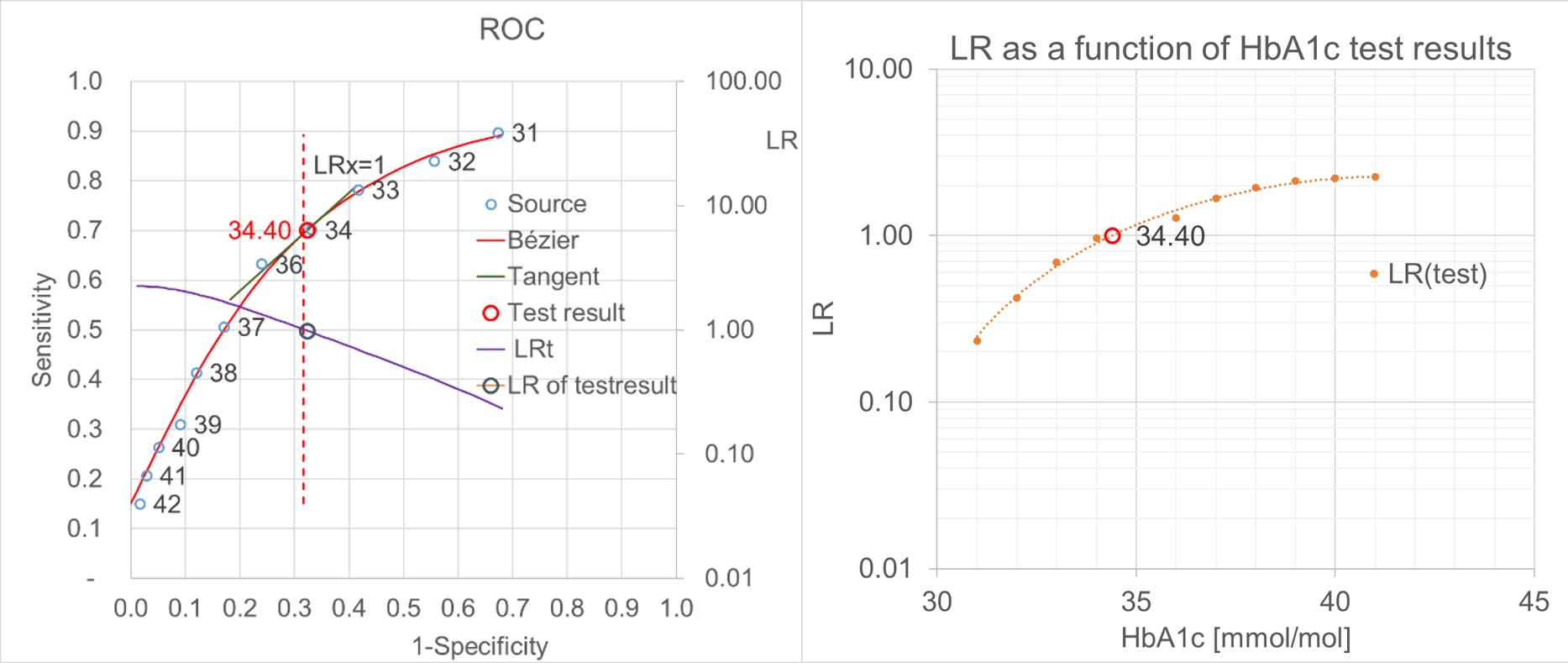

Supplement: Supplementary Figure 1 — ROC curves with test result values (o) corresponding to the individual points of the curve (left) and LR as a function of test results (right) as calculated by the Bézier curves method (29). Test results with LR=1 are indicated in red. (A) Fasting capillary blood glucose as a screening test for diabetes (40). (B) D-dimer testing for suspected pulmonary embolism in outpatients (41). (C) PSA testing Gleason grade ≥7 vs Gleason grade <7 or no cancer (42). (D) HbA1c Test as a Tool in the Diagnosis of Gestational Diabetes Mellitus (43). [file DataSheet_1.zip › Supplementary Figure 1A.jpg]

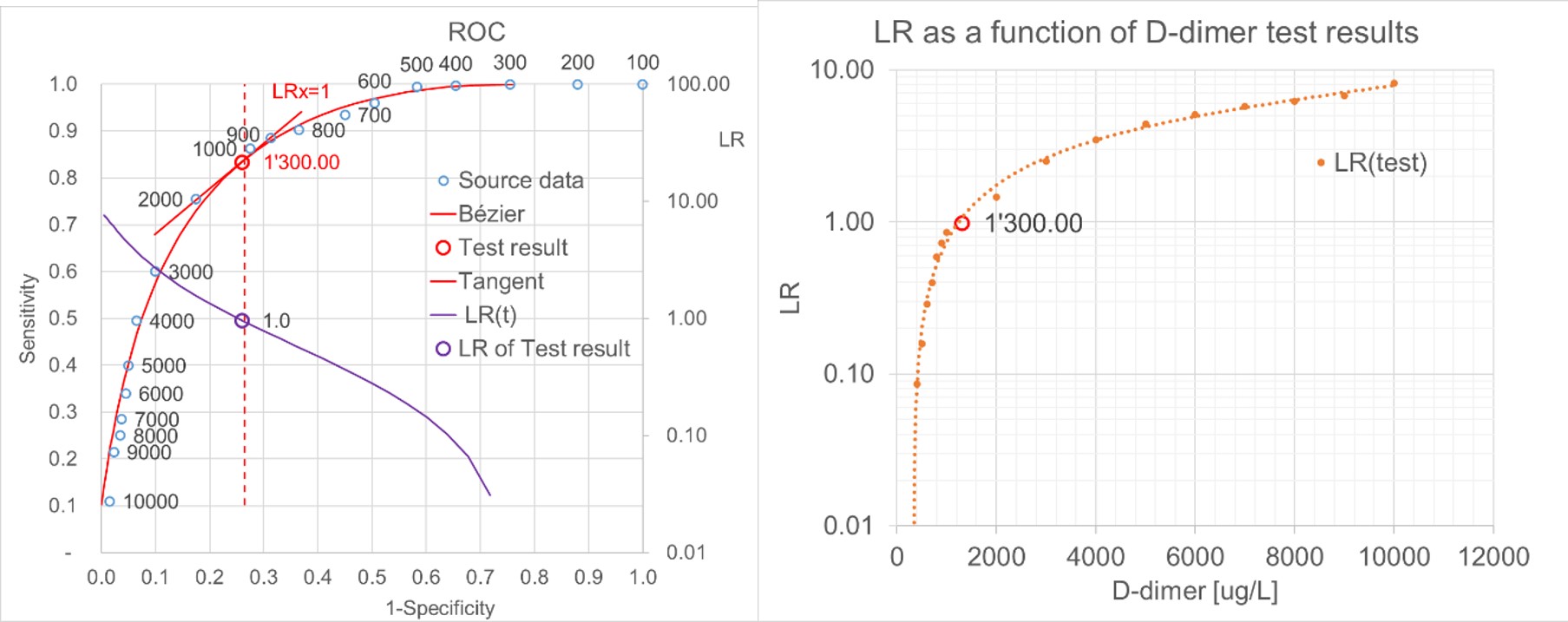

Supplement: Supplementary Figure 1 — ROC curves with test result values (o) corresponding to the individual points of the curve (left) and LR as a function of test results (right) as calculated by the Bézier curves method (29). Test results with LR=1 are indicated in red. (A) Fasting capillary blood glucose as a screening test for diabetes (40). (B) D-dimer testing for suspected pulmonary embolism in outpatients (41). (C) PSA testing Gleason grade ≥7 vs Gleason grade <7 or no cancer (42). (D) HbA1c Test as a Tool in the Diagnosis of Gestational Diabetes Mellitus (43). [file DataSheet_1.zip › Supplementary Figure 1B.jpg]

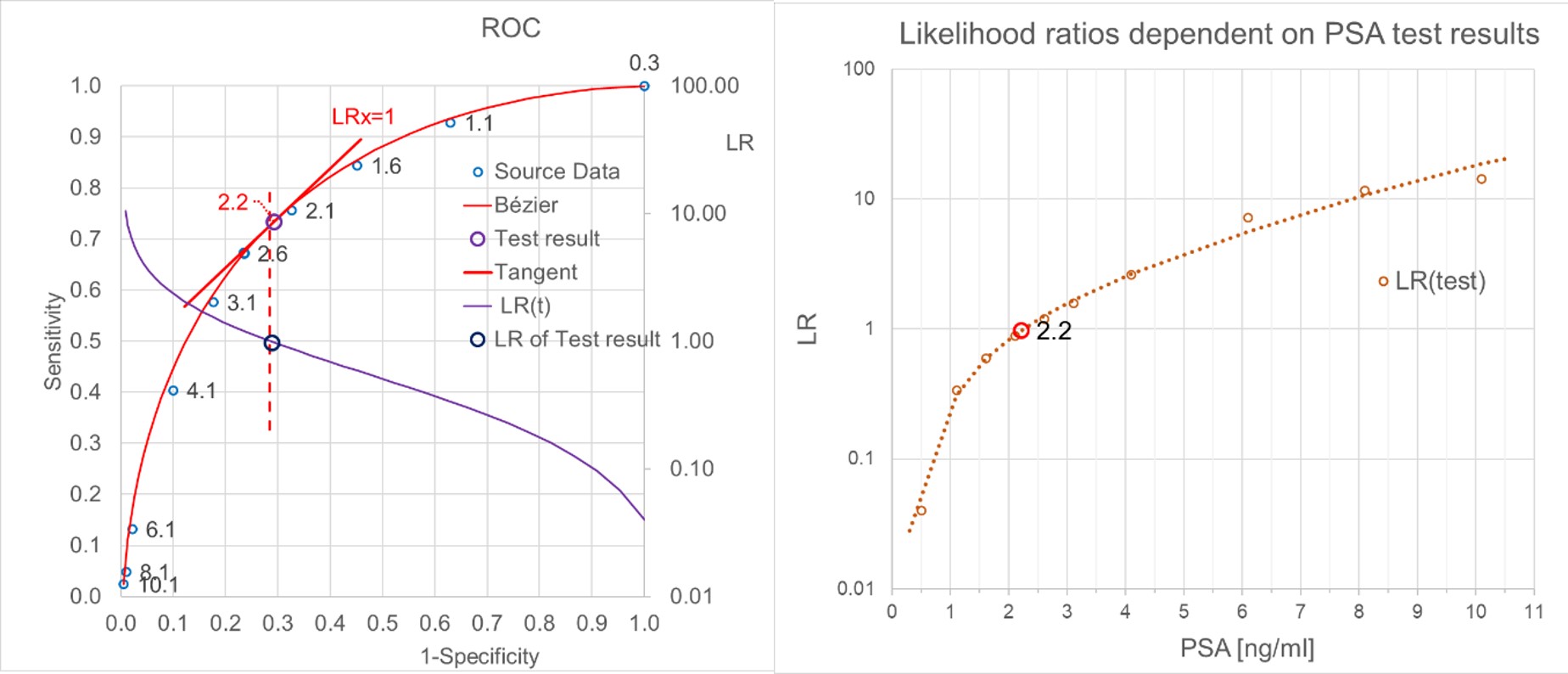

Supplement: Supplementary Figure 1 — ROC curves with test result values (o) corresponding to the individual points of the curve (left) and LR as a function of test results (right) as calculated by the Bézier curves method (29). Test results with LR=1 are indicated in red. (A) Fasting capillary blood glucose as a screening test for diabetes (40). (B) D-dimer testing for suspected pulmonary embolism in outpatients (41). (C) PSA testing Gleason grade ≥7 vs Gleason grade <7 or no cancer (42). (D) HbA1c Test as a Tool in the Diagnosis of Gestational Diabetes Mellitus (43). [file DataSheet_1.zip › Supplementary Figure 1C.jpg]

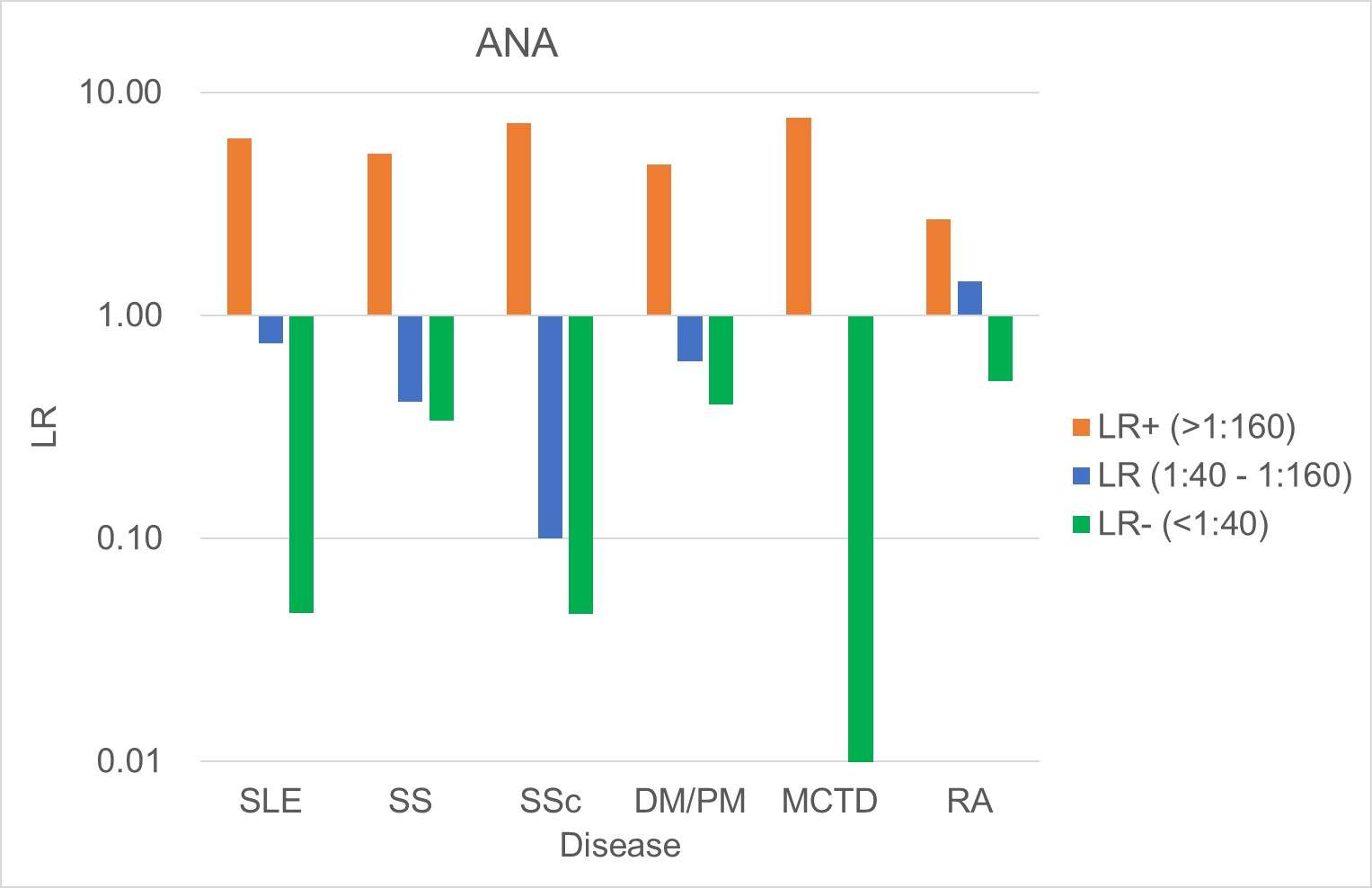

Supplement: Supplementary Figure 1 — ROC curves with test result values (o) corresponding to the individual points of the curve (left) and LR as a function of test results (right) as calculated by the Bézier curves method (29). Test results with LR=1 are indicated in red. (A) Fasting capillary blood glucose as a screening test for diabetes (40). (B) D-dimer testing for suspected pulmonary embolism in outpatients (41). (C) PSA testing Gleason grade ≥7 vs Gleason grade <7 or no cancer (42). (D) HbA1c Test as a Tool in the Diagnosis of Gestational Diabetes Mellitus (43). [file DataSheet_1.zip › Supplementary Figure 2.jpg]
